# Anatomic Variation of the Lateral Sinus in Patients With Idiopathic Intracranial Hypertension: Delineation With Black-Blood Contrast-Enhanced MRI

**DOI:** 10.3389/fneur.2021.715857

**Published:** 2021-11-25

**Authors:** Yu Tian, Zhe Zhang, Jing Jing, Kehui Dong, Dapeng Mo, Yilong Wang

**Affiliations:** ^1^Department of Neurology, Beijing Tiantan Hospital, Capital Medical University, Beijing, China; ^2^Chinese Institute for Brain Research, Beijing, China; ^3^China National Clinical Research Center for Neurological Diseases, Beijing, China; ^4^Advanced Innovation Center for Human Brain Protection, Capital Medical University, Beijing, China; ^5^Beijing Key Laboratory of Translational Medicine for Cerebrovascular Disease, Beijing, China; ^6^Department of Interventional Neurology, Beijing Tiantan Hospital, Capital Medical University, Beijing, China

**Keywords:** pseudotumor cerebri syndrome, Idiopathic intracranial hypertension (IIH), cerebral venous sinus stenosis (CVSS), arachnoid granulation, black-blood magnetic resonance imaging (BB-MRI)

## Abstract

**Objectives:** The purpose of this study was to describe the peculiar anatomic variations in the lateral sinus and analyze the patterns of cerebrospinal fluid (CSF) drainage by using high-resolution (HR) black-blood (BB) contrast-enhanced magnetic resonance imaging (MRI) in patients with idiopathic intracranial hypertension (IIH).

**Methods:** Total 33 IIH patients who were found cerebral venous sinus stenosis (CVSS) by MR venography (MRV) were enrolled in this study. HR-BB contrast-enhanced MRI was used to assess the features of anatomical variations in transverse sinus and sigmoid sinus. The development of bilateral sinuses was firstly evaluated, including unilateral hypoplasia with contralateral dominance or bilateral balanced development. Then, four kinds of anatomical variations were eventually recorded, including circumscribed stenosis, arachnoid granulation (AG), fibrous septum (FS), and brain herniation (BH) into dural venous sinus (DVS).

**Results:** Bilateral venous drainage dysfunction was found in 30(90.9%) patients, whereas only 3(9.1%) patients presented unilateral venous drainage dysfunction. There was no difference in clinical symptoms between the two groups. The most common case is hypoplasia in unilateral sinus combined with anatomic variation in the contralateral dominant transverse sinus such as AG and BH into DVS. Total of 52 anatomic variations were finally found in bilateral sinuses in 33 enrolled patients, including 19(36.5%)AGs, 12(23.1%)FS, 7(13.5%) BH into DVS and 14(26.9%) circumscribed stenoses. Moreover, 41(62.1%) lateral sinuses showed enhancement in T1-weight-enhanced MRI.

**Conclusions:** Patients with CVSS almost had CSF outflow disorders, whatever bilateral equalization or unilateral hypoplasia with contralateral dominance. Four types of main anatomic variations, including circumscribed stenosis, AG, FS, and BH into DVS, caused venous reflux obstruction by elevating the intracranial press (ICP).

## Introduction

Idiopathic intracranial hypertension (IIH), also called pseudotumor cerebri-like syndrome (PTCS) (1), is a disorder characterized by raised intracranial pressure (ICP) with normal cerebrospinal fluid (CSF) composition. Clinically, IIH manifests with headache and papilledema and often accompanies by visual disturbance. IIH is typically seen in women who are obese and of childbearing age, with a worldwide incidence of 0.5–2 per 100,000 people per year in the general population (2). The etiology and pathogenic mechanism of IIH is still unclear and needs to be investigated. Several hypotheses have been proposed, including CSF overproduction, insufficient CSF drainage, and alterations in cerebral blood volume, as well as endocrine disorders (1, 3, 4).

In the past, due to the limitations of imaging methods, the relationship between cerebral venous sinus stenosis (CVSS) and IIH was underestimated (5, 6). Recently, CVSS has increasingly been recognized as a relevant factor in the pathogenesis of PTCS. CVSS is reported to occur approximately over 93% in patients with IIH, significantly higher than the general population (7–9). Moreover, Bilateral CVSS is reported in nearly 65–100% patients with IIH (10). However, the nature and cause of stenosis are currently unclear. Some anatomic variations, including circumscribed stenosis, hypoplasia, arachnoid granulation (AG), fibrous septum (FS), and brain herniation (BH), have been reported present in dural venous sinuses(DVSs) of patients with IIH which may lead to elevating ICP (8, 9, 11, 12). There are very few published data describing the characteristics of stenosis and the distribution of anatomic variations in IIH patients with CVSS.

High-resolution (HR) black-blood (BB) contrast-enhanced MRI (MRI), a high-resolution new magnetic resonance imaging technique for inhibiting flow blood signal, has gained broad interest. Several studies have confirmed high feasibility and accuracy for detecting thrombus, atherosclerotic plaques, AG and vasculitis (13–18). Hence, in order to better understand the nature of stenosis and pathogenesis of IIH, we try to describe the anatomic variation in stenosis of DVSs and analyze the asymmetry of CSF drainage in patients with IIH by using HRBB contrast-enhanced MRI.

## Subjects and Methods

### Study Population

Patients who were diagnosed with IIH at Beijing Tiantan Hospital of the Capital Medical University were consecutively enrolled in our study between November 2018 and January 2020. Inclusion criteria: (1) age range from 16 to 80 years; (2) diagnosis of IIH according to the modified Dandy criteria (19), including signs and symptoms of increased ICP, absence of localizing findings on neurologic examination, normal neurodiagnostic studies except for evidence of increased CSF pressure (>200 mm H_2_O) and abnormal neuroimaging except for empty sella turcica, optic nerve sheath with filled out CSF spaces, and smooth-walled non-flow-related venous sinus stenosis or collapse should lead to another diagnosis; (3) definite radiographic diagnosis of CVSS by MRV or computerized tomography venography (CTV) or Digital Substraction Angiography (DSA). Exclusion criteria including general contraindications to MR examination like claustrophobia, severe contrast allergy and severe hepatic and renal dysfunction.

### Clinical Data

The clinical data of patients were collected by the clinicians after admission, including age, gender, body mass index(BMI), clinical symptoms(headache, visual symptom, tinnitus, and papilledema) and symptom duration, and treatment. All patients had a lumbar puncture performed by a bedside clinician and required the CSF opening pressure. Ophthalmological assessment is used to assess the presence of papilledema.

### Black-Blood Magnetic Resonance Imaging

All MR images were acquired on a 3.0T MRI scanner (Ingenia CX; Philips, Best, the Netherlands), using a 32-channel phased-array head coil. The MRI protocol includes 3D MR venography (MRV), 3D T1w structural imaging using magnetization-prepared rapid gradient-echo sequence (MPRAGE), 3D T1w black-blood imaging with motion-sensitized driven-equilibrium (MSDE) prepulse (20) and 3D T2w black-blood imaging with MSDE prepulse. 3D T1w structural imaging and 3D T1w black-blood imaging were acquired before and after the gadolinium-based contrast agent (gadopentetate dimeglumine, 0.1 mmol/kg) injection. The detailed scan parameters are listed in the Table 1.

**Table 1:** Detailed scan parameters of the MRI protocol.

**Scan name**	**Sequence**	**Orientation**	**FOV (AP × RL × FH, mm^**3**^)**	**TE/TR (ms)**	**Reconstruction voxel size(mm^**3**^)**	**Other**
MRV	3D Phase contrast (PC)	Transverse	230 × 180 × 200	3.4/13.9	0.53 × 0.53 × 0.80	
T1w structural	3D MPRAGE	Sagittal	240 × 196 × 240	3.0/6.4	1 × 1 × 1	Acquired before and after contrast injection
T1w black-blood	3D TSE (Turbo Spin-echo)	Sagittal	220 × 160 × 240	21/800	0.7 × 0.7 × 0.7	Acquired before and after contrast injection
T2w black-blood	3D TSE (Turbo Spin-echo)	Sagittal	220 × 160 × 240	194/2500	0.7 × 0.7 × 0.7	

### Image Analysis

All black-blood MRI images were randomized and presented to 2 independent readers with more than 5 years of experience in reading. The readers were not involved with the diagnostic or therapeutic management of the patients and were blinded to clinical information and conventional imaging data on which the diagnosis of IIH was based. And a third reader with 10 years in experience of reading was involved to resolve any disputes.

Bilateral sinuses, including transverse sinus, sigmoid sinus and transverse-sigmoid conjunction, were included in the evaluation. The development of bilateral sinuses was firstly evaluated, including unilateral hypoplasia with contralateral dominance or bilateral balanced development. A hypoplastic sinus was defined as a sinus that is 40% smaller in average caliber than the contralateral sinus which could be called “dominant” (9). Then, 4 types of anatomic variations were evaluated in bilateral sinuses. Circumscribed stenosis was defined as an abrupt 40% reduction in caliber of the vessel (9). AG is an invagination of the arachnoid membrane that perforates gaps in the dura and protrude into the lumen of the dural sinus (10). Intra-sinus BH, different from AG, is an invagination of brain parenchyma into the cerebral dural venous sinus (12). FS had a curved smooth shape that conformed to that of the sinus, and typically was oriented along the long axis of the sinus and sometimes extended the whole course of the sinus (11, 21).

### Statistical Analysis

Categorical variables were presented as percentages and continuous variables as mean with SD or median with IQR. Statistical analyses of categorical variables were carried out using χ2 and Fisher exact tests as appropriate. Statistics of means were carried out using the unpaired Student *t*-test, both with and without equal variance (Levene test) as necessary, and Wilcoxon rank sum test when variables were not normally distributed. *P* values of ≤ 0.05 were considered statistically significant.

## Results

### Patients Characteristics

According to the inclusion and exclusion criteria, a total of 33 patients who had an average age of (34.09 ± 10.72) years, with 11(33.3%) male and 22(66.7%) female, were consecutively enrolled in our study. The typical clinical symptoms were visual symptom (including impaired vision, visual field defect and diplopia), papilledema, headache and pulsatile tinnitus in 24(72.7%), 23(69.7%), 22(66.7%) and 5(15%) patients, respectively. Symptom duration was (11.3 ± 17.5) month. Among these, 22(66.7%) patients received drug treatment, such as acetazolamide, mannitol and furosemide, while 14(42%) patients received endovascular treatment with stent placement (including 3 cases of drug treatment failure). The results of the clinical data are summarized in Table 2.

**Table 2:** Clinical features of 33 enrolled patients with IIH.

**Case No**.	**Gender (Male or Female)**	**Age (years)**	**BMI (Kg/m^**2**^)**	**Symptoms**				**Symptom duration (months)**	**ICP (mm H_**2**_0)**	**Treatment**	
				**H**	**VS**	**T**	**PA**			**Drug**	**Stenting**
1	M	34	26.23	-	-	-	-	24	240	+	-
2	M	50	27.06	-	+	-	+	1	260	+	-
3	F	31	28.96	+	-	-	-	4	330	+	+^*^
4	F	51	20.70	+	-	-	-	6	330	+	-
5	F	55	24.77	-	+	-	+	1	310	+	-
6	F	36	22.96	+	-	+	-	48	380	+	-
7	M	26	27.76	+	+	-	-	60	265	+	+^*^
8	F	46	29.27	-	-	+	-	36	250	-	+
9	F	24	20.20	+	-	+	-	60	>330	+	-
10	F	21	25.95	-	+	-	+	0.5	>330	-	+
11	M	47	24.69	+	+	-	-	2	245	+	-
12	M	44	21.22	-	+	-	+	6	320	-	+
13	F	46	31.64	+	+	-	+	1	285	-	+
14	F	41	32.30	+	+	-	+	4	>330	+	-
15	F	20	34.37	+	-	-	+	6	>330	+	-
16	F	36	39.54	+	+	-	+	1	>330	-	+
17	M	36	29.05	+	+	-	+	4	>330	+	-
18	M	30	35.03	+	+	-	+	1	>330	+	-
19	F	35	20.83	+	+	-	+	2	255	+	-
20	F	21	22.66	-	+	-	+	2	315	+	-
21	M	30	33.22	-	+	-	-	1	265	+	-
22	M	16	27.01	+	+	-	+	2	200	+	-
23	F	38	24.03	-	+	-	+	1	310	-	+
24	F	50	25.33	+	+	+	+	36	>330	-	+
25	F	30	19.78	+	-	-	+	10	300	+	-
26	F	16	26.17	+	-	-	-	0.5	400	+	-
27	F	36	28.04	+	+	+	+	1	240	+	-
28	F	19	18.82	+	+	-	+	4	310	+	-
29	F	42	34.48	-	+	-	+	36	510	-	+
30	F	23	28.91	+	+	-	+	4	450	-	+
31	M	39	25.33	+	+	-	+	0.5	320	-	+
32	F	27	25.39	-	+	-	+	3	280	+	+^*^
33	M	29	29.07	+	+	-	+	3	325	-	+

*BMI, body mass index; H, headache; ICP, intracranial pressure; T, tinnitus; PA, papilledema; V, visual symptom; +, yes; -, no*.

* *drug treatment failure*.

### The Anatomy of Bilateral Sinuses

**Figure 1:**
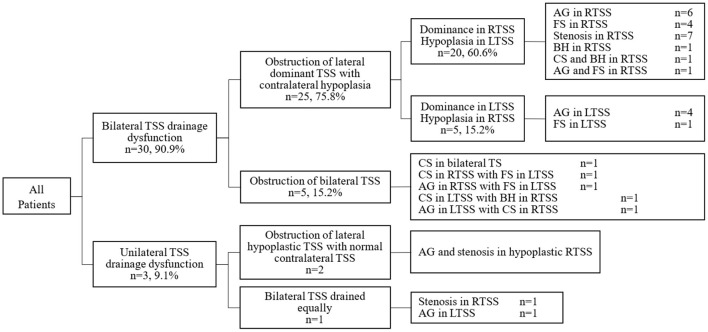
The anatomic variations and drainage patterns in 33 enrolled patients with IIH.

We finally evaluated the anatomy of bilateral sinuses and cerebral venous outflow from 33 enrolled patients with CVSS by HRBB contrast-enhanced MRI (Figure 1; Table 3). Bilateral venous drainage dysfunction was found in 30(90.9%) patients. Among these, obstructions of lateral dominant transverse sinus drainage with contralateral hypoplasia were presented in 25(75.8%) patients. The SSS preferentially drained to the right transverse sinus with the left transverse sinus hypoplasia in 5 cases (15.2%) and to the left transverse sinus with right transverse sinus hypoplasia in 20 patients (60.6%). In these 25 patients, 4 types of anatomic variations, such as AG, BH, FS, and local stenosis, presented in the dominant sinuses which led to the blockage of CSF drainage. In another 5(15.2%) cases in which the SSS drained equally to bilateral sinuses, bilateral sinuses presented anatomic variations and were blocked simultaneously.

**Table 3:** The anatomy of bilateral sinuses in 33 enrolled patients with IIH.

**Case No**.	**TOF-MRV**		**HR-BB contrast-enhanced MRI**					
	**Left transverse sinus**	**Right transverse sinus**	**Left transverse sinus**			**Right transverse sinus**		
			**Anatomical variation**	**Lesion enhanced**	**Wall enhanced**	**Anatomical variation**	**Lesion enhanced**	**Wall enhanced**
1	Hypoplasia	Stenosis	Hypoplasia&AG	Yes	Yes	AG	Yes	No
2	Hypoplasia	Normal	Hypoplasia&AG	Yes	Yes	AG	No	No
3	Hypoplasia	Stenosis	Hypoplasia&AG	Yes	Yes	AG	Yes	No
4	Hypoplasia	Stenosis	Hypoplasia&AG	Yes	Yes	AG	Yes	Yes
5	Hypoplasia	Normal	Hypoplasia&AG	Yes	Yes	FS	No	No
6	Hypoplasia	Normal	Hypoplasia&FS	No	Yes	FS	No	Yes
7	Hypoplasia	Stenosis	Hypoplasia&FS	Yes	Yes	AG	Yes	Yes
8	Hypoplasia	Stenosis	Hypoplasia&BH	Yes	Yes	CS	No	Yes
9	Hypoplasia	Normal	Hypoplasia&BH	No	No	CS	No	No
10	Hypoplasia	Stenosis	Hypoplasia	No	Yes	CS&BH	No	No
11	Hypoplasia	Stenosis	Hypoplasia	No	Yes	AG&FS	No	No
12	Hypoplasia	Stenosis	Hypoplasia	No	No	AG	No	No
13	Hypoplasia	Stenosis	Hypoplasia	No	Yes	BH	No	Yes
14	Hypoplasia	Stenosis	Hypoplasia	No	No	FS	No	No
15	Hypoplasia	Stenosis	Hypoplasia	No	Yes	CS	No	No
16	Hypoplasia	Stenosis	Hypoplasia	No	No	CS	No	No
17	Hypoplasia	Stenosis	Hypoplasia	No	No	CS	No	Yes
18	Hypoplasia	Stenosis	Hypoplasia	No	Yes	CS	No	Yes
19	Hypoplasia	Stenosis	Hypoplasia	No	Yes	CS	No	No
20	Hypoplasia	Normal	Hypoplasia	No	Yes	FS	No	Yes
21	Stenosis	Hypoplasia	FS	No	No	Hypoplasia&FS	No	Yes
22	Normal	Hypoplasia	AG	Yes	Yes	Hypoplasia&FS	No	No
23	Stenosis	Hypoplasia	AG	No	Yes	Hypoplasia&BH	Yes	Yes
24	Stenosis	Hypoplasia	AG	Yes	Yes	Hypoplasia	No	Yes
25	Stenosis	Hypoplasia	AG	Yes	Yes	Hypoplasia	No	Yes
26	Stenosis	Stenosis	FS	No	Yes	CS	No	No
27	Normal	Stenosis	FS	Yes	Yes	AG	Yes	Yes
28	Stenosis	Normal	CS	No	No	CS	No	No
29	Stenosis	Stenosis	CS	No	Yes	BH	Yes	Yes
30	Stenosis	Stenosis	AG	Yes	Yes	CS	No	No
31	Hypoplasia	Normal	BH	Yes	Yes	Normal	No	No
32	Normal	Stenosis	AG	Yes	Yes	Normal	No	No
33	Normal	Stenosis	Normal	No	No	CS	No	No

*AG, arachnoid granulation; FS, fibrous septum; CS, circumscribed stenosis; BH, brain herniation in to DVS*.

Only unilateral venous drainage disorder was found in 3(9.1%) patients. In 2 cases which bilateral TS grew equally, bilateral TS drainage dysfunction was caused by AG or circumscribed stenosis. And obstruction of hypoplastic TS with normal contralateral dominant TSS were presented in 1 case.

According to the cerebral venous outflow pattern, patients were divided two group: patients with bilateral TSS drainage dysfunction (*n* = 30) and patients with unilateral TSS drainage dysfunction (*n* = 3). The baseline characteristics, clinical presentation, CSF opening pressure grade and therapeutic procedures between the two groups showed no statistical difference. Details are displayed in Table 4.

**Table 4:** Clinical features of different cerebral venous outflow pattern in 33 enrolled patients with IIH.

	**Group A (*n* = 30)**	**Group B (*n* = 3)**	***P* value**
Age, years, median (IQR)	35.5(23.8–44.5)	29(27-)	0.70
Female, *n* (%)	21(95.5)	1(33.3)	0.25
BMI, Kg/m^2^, median (IQR)	26.6(22.9–29.9)	25.4(25.3-)	0.89
Symptoms, n (%)			
Headache	20(66.7)	2(66.7)	1.00
Visual symptom	21(70.0)	3(100.0)	0.55
Tinnitus	5(100.0)	0(0.0)	1.00
Papilledema	20(66.7)	3(100.0)	0.54
Duration, months, median (IQR)	3(1–8)	3(0.5-)	0.35
ICP grade, *n* (%)			0.62
(200~250) mm H_2_0	4(13.3)	0(0.0)	
(250~300) mm H_2_0	6(20.0)	1(3.3)	
≥300 mmH_2_0	20(66.7)	2(66.7)	
Treatment, *n* (%)			
Drug	21(70.0)	1(33.3)	0.25
Stenting	11(36.7)	3(100.0)	0.067

*Group A, Patients with bilateral TSS drainage dysfunction; Group B, Patients with unilateral TSS drainage dysfunction; BMI, body mass index; ICP, intracranial pressure*.

### Anatomic Variations

**Figure 2:**
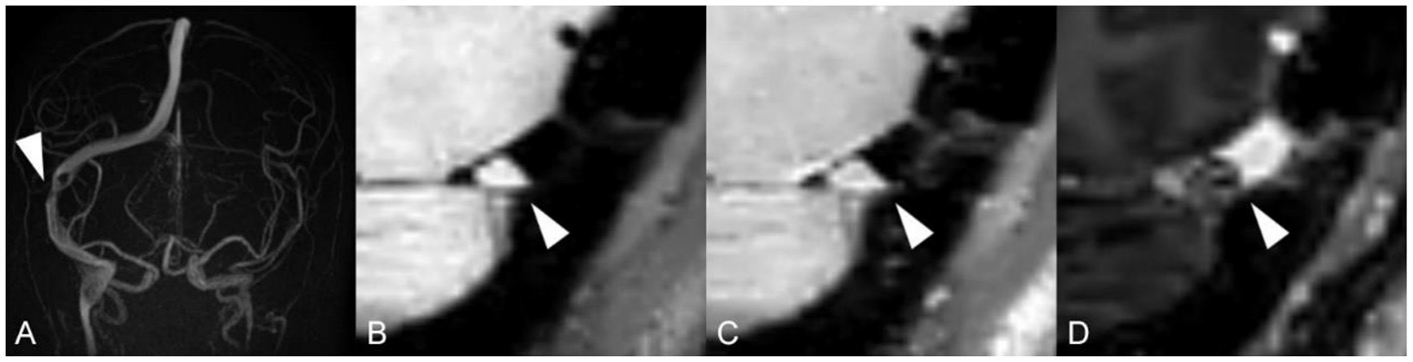
Images reveal arachnoid granulation in a 51-year-old woman with headache. **(A)** MRV shows stenosis on RTS with left occlusion/ hypoplasia; **(B)** T1w black-blood image shows an oval arachnoid granulation that is isointense to brain parenchyma; **(C)** T1w black-blood contrast-enhanced image shows a an oval arachnoid granulation and an enhanced dural venous sinus wall; **(D)** T1w contrast-enhanced image shows a moth-eaten filling defect.

**Figure 3:**
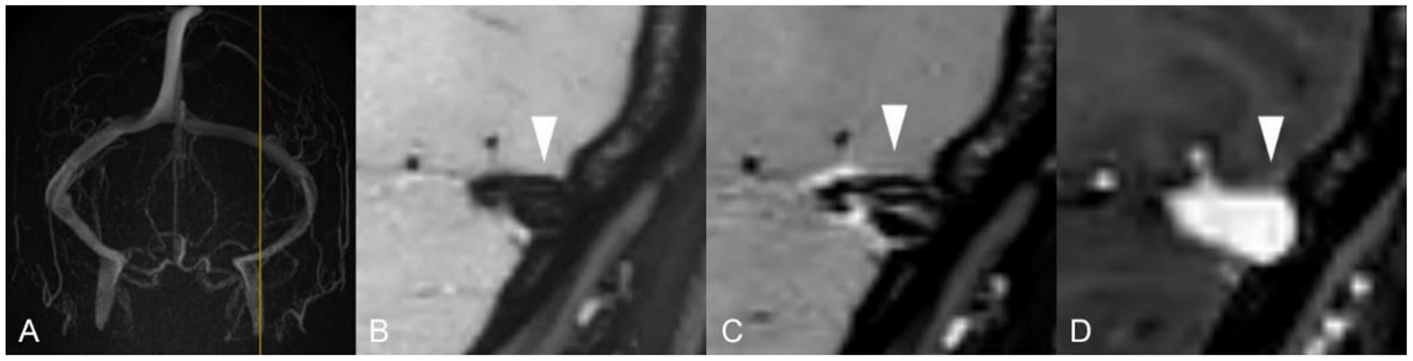
Images reveal a fibrous septum in 36-year-old woman with tinnitus and diplopia. **(A)** MRV shows normal venous sinus; **(B, C)** T1w black-blood image and T1w black contrast-enhanced image shows fibrous septum in DVS lumen; **(D)** T1w contrast-enhanced image shows a normal lumen without any filling defect.

**Figure 4:**
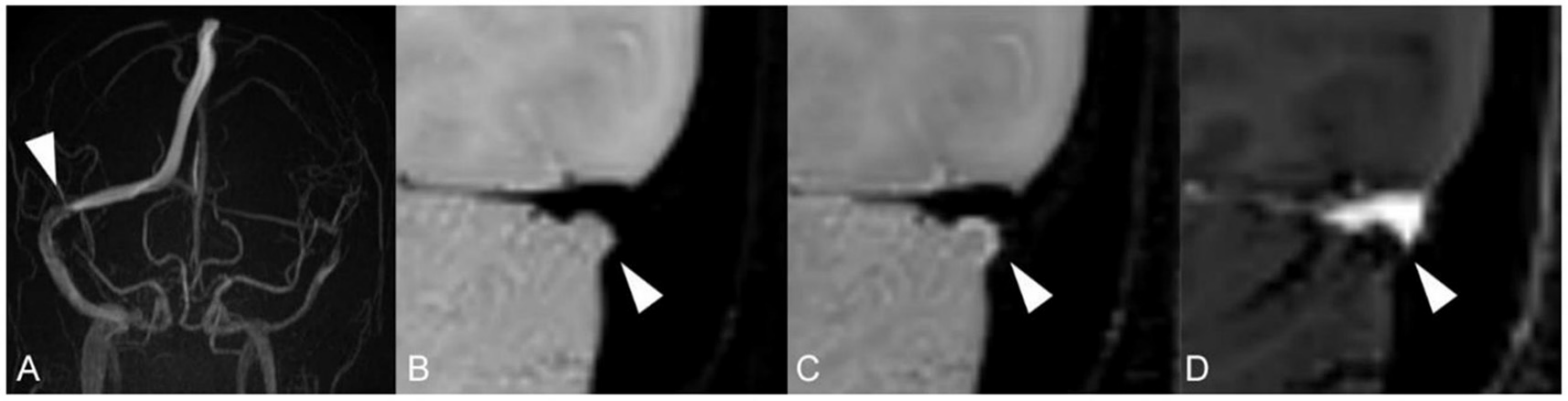
Images reveal intra-sinus brain herniation in 46-year-old woman with headache and blurred vision. **(A)** MRV shows occlusion/ hypoplasia on LTS with normal RTS; **(B)** T1w black-blood image shows a small herniation of cerebellum parenchyma with surrounding CSF into RTS that was isointense on T1w; **(C)** T1w black-blood contrast-enhanced image shows a hyperintense margins surrounding the brain herniation; **(D)** T1w enhanced image shows a focal filling defect.

**Figure 5:**
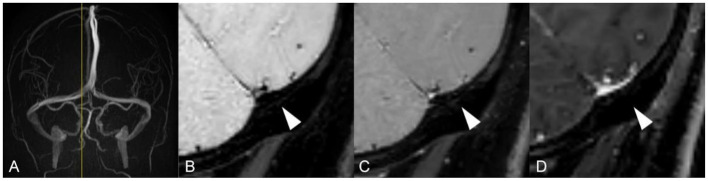
Images reveal a stenosis in 29-year-old man with intracranial hypertension symptom. **(A)** MRV shows normal venous sinus; **(B–D)** T1w black-blood image, T1w black-blood contrast-enhanced image and T1w contrast-enhanced image show thin cava vascular lumen.

Unlike standard angiography such as CTV, MRV or DSA, which displays external morphology of the stenotic segment in the dural sinus, HR-BB contrast-enhanced MRI can directly reveal intra-sinus characteristics to better identify the nature of the lesions. Total 66 lateral sinuses from 33 enrolled patients were finally estimated (Table 1), and 52 anatomic variations were found. According to the characteristics of CVSS and previous studies, four types of anatomic variations that could be visualized were arachnoid granulations (19, 36.5%, Figure 2), fibrous septa (12, 23.1%, Figure 3), brain herniations into DVS(7, 13.5%, Figure 4) and circumscribed stenoses(14, 26.9%, Figure 5; Table 3). Interestingly, two types of anatomical variations could present simultaneously on one sinus, such as AG combined with FS. On the contrast-enhanced T1-weighted MR images, a total 41(62.1%) transverse sinuses showed enhancement, including 19(28.8%) both lesions and walls enhanced, 2(3.0%) lesions enhanced, 20(30.3%) walls of the sinuses enhanced. As far as we know, these four types of anatomical variations of images represent the first simultaneous *in vivo* description of intraluminal anomalies related to cerebral venous stenosis by HRBB-MRI.

## Discussion

The presence of CVSS was the “usual” finding in the intracerebral venous system of PTCS. Unilateral and bilateral CVSS was reported to occur in approximately 18 and 6% of the general population, respectively (9), whereas over 90% in patients with IIH (22). Previous studies showed that venous sinus stenting in patients with IIH has achieved a good clinical effect to relive symptoms, which supports the hypothesis venous sinus stenosis is one of the risk factors of IIH (23, 24). However, the feature and the specific anatomic causes of the narrowing is relatively unexplored. To investigate the prevalence and characteristics of “stenosis” and hypoplasia, we enrolled 33 patients who have a clinical diagnosis of IIH and present with CVSS verified by MRV or CTV. HR black-blood contrast-enhanced MRI was used to evaluate the CSF drainage of bilateral transverse-sigmoid sinuses (BTSS) and further describe anatomic variations of stenosis located in the lateral sinus.

The anatomy of lateral sinuses and CSF outflow are difficult to assess, seeming to present a wide variation in normal situation. Christopher et al. investigated the prevalence of stenosis and hypoplasia of DVSs in 355 enrolled patients, which was believed to represent the largest investigation in the general population (9). They found the prevalence of unilateral transverse sinus stenosis or hypoplasia was 33%, while the prevalence of BTSS was 5% and the prevalence of unilateral stenosis with contralateral hypoplasia was 1% (9). As well as, Ayanzen et al. found 31% of cases with normal MRI found flow gaps in the lateral sinuses, with 90% of these occurring in the non-dominant transverse sinus and 10% in the codominant transverse sinuses (25). Transverse sinuses were found to be right, left, and codominant in 59%, 25%, and 16% of the cases examined, respectively (25). Riggeal et al. found transverse sinus stenosis is common, if not universal, among patients with IIH, and is almost always bilateral (46/51, 90%) (26).

In our study, we finally found 30(90.9%) patients presented bilateral venous drainage dysfunction, whereas only 3(9.1%) patients presented unilateral venous drainage dysfunction. The most common situation is that anatomical lesion present in the dominant transverse sinus combined with contralateral hypoplasia. Transverse sinuses were found to be right dominant, left dominant, and codominant in 60.6%, 15.1%, and 24.4% of the cases examined, respectively. In patients with hypoplasia in the unilateral transverse-sigmoid sinus, cerebrospinal fluid drainage is performed through the contralateral transverse sigmoid sinus to maintain stable intracranial pressure. Once the contralateral sinus presents stenosis which obstruct intra-sinus cavity, CSF reflux will be impaired and the patient will present with clinical symptoms of intracranial hypertension such as headache, nausea, vomiting, and papilledema. We didn't found the difference of clinical characteristics between patients with bilateral CSF drainage dysfunction and patients with unilateral CSF drainage dysfunction, further studies are needed in the future.

Previous studies indicated that the presence of AG, septum, BH into DVS, and other local anatomic stenosis is associated with the etiology of IIH. Total 52 anatomic variations were finally detected in the present study, including 19(36.5%) arachnoid granulations, 12(23.1%) fibrous septa into DVSs, 7(13.5%) brain herniations, and 14(26.9%) circumscribed stenoses. Arachnoid granulations are pseudopodial anatomic structures, which are invaginations of the arachnoid membrane, perforate gaps in the dura, and protrude into the lumen of the dural sinus (11, 27). In routine CT and MRI, AGs are demonstrated as well circumscribed structures that have the same density or signal intensity as CSF relative to brain parenchyma (27, 28). Christopher et al. found that AGs were identified in 70% of patients with unilateral stenosis and only 18% of patients with no transverse sinus stenosis had AGs (9). In our study, 15(45.5%) enrolled patients had AG in their lateral sinuses. We also found in 25 cases which had obstruction of lateral dominant TSS with contralateral hypoplasia, AGs were detected in the dominant lateral sinuses of 11(45.5%) cases. And AG also appeared in the hypoplastic lateral sinus. In addition, 4 patients had AG in the bilateral transverse sinuses. Deserved to be mentioned, in many cases where AGs were detected in DVS, it was the growth of AGs that caused the potential impediment of outflow through the TSS. AGs form as ICP increases, and increasing AGs could lead to the impediment of outflow of DVS, which in turn leads to elevated ICP (9, 29). It could lead to a positive feedback mechanism.

Intra-sinus BH was always confused with AG, however, with improved awareness and growing utilization of new imaging techniques, BH into DVS has been considered as an independent anatomic variation that has developed spontaneously or been induced by elevated ICP or underlying AG (12, 30, 31). In Bilal et al. study (12) and Greta et al. study (30), BHs with surrounding CSF into the DVSs were not associated with any symptoms. However, Coban et al. (32) and Karatag et al. (33) showed that BH into DVS may have been related to dizziness, a sensation of pressure in ear, and headache. In our study, intra-sinus BHs were found in 7(21.2%) patients who had clinical symptoms such as headache and visual loss. The incidence of intra-sinus BHs in patients with IIH is significantly higher than it in the general population (020/6160, 0.32%) (12). Thus, we can only speculate that increased ICP may be a predisposing condition or consequence of intra-sinus BH. Now there are not any comprehensive evaluations about the incidence, etiology, pathology, imaging features, and clinical features of BH into DVS, more research is needed for that in the future.

Previous studies have proved the presence of fibrous septa in patients with IIH by dissecting human cadavers, potentially as a result of the merger of the embryonic venous plexus into the DVS during early fetal development (21). In Liang et al. study, approximately 90% of patients had one or more septa in the straight sinus (11). However, in our study, the septa are found in the transverse sinus. Fibrous septum typically appeared as multiple thin parallel structures in the transverse sinus and was sometimes oriented along the long axis of the sinus. A total of 12 FSs were detected in the lateral sinus from 33 enrolled patients, although it is not clear whether fibrous septa were the cause of the clinical symptoms in patients with IIH. Lansley et al. showed the septa were more frequently present in patients with IIH, however, there were no support for the proposed pathophysiologic mechanism that septa are directly responsible for pulsatile tinnitus in IIH (34). Subramaniam et al. suggested the presence of fibrous septum might not only narrow the lumen anatomically but also contribute to turbulent flow that may arise ICP and cause clinical symptoms (21).

Some studies indicated that sinus stenosis leading to raised ICP found in subjects with IIH was due to a collapse of the sinus walls caused by elevated ICP (35, 36). In our study, 26.9% of anatomic variations showed up as a sudden thinning of the sinus cavity, called circumscribed stenosis. The cause of these stenoses in patients with IIH remains uncertain, and there is no answer whether these stenoses will get worse or alleviative. Rohr et al. reported venous outflow stenosis had an initial resolution of symptoms after insertion of a stent into the transverse sinus in a patients with IIH, but the symptoms recurred and a restenosis was noted just upstream from the stent (37). Guillaume et al. reported a case about a 16-year-old boy with bilateral papilledema and raised ICP (38). After depletive lumbar puncture, the size of AGs in both transverse sinuses decreased and sinus lumen stenosis resolved (38), suggesting stenosis may be the consequence of the pathological process of IIH rather than a cause of the disorder.

It is important to note that the anatomic variations are not mistaken for pathology, which may be “normal”, innate, or secondary to elevated ICH. However, some studies have been indirectly implicated that anatomic variations accompanied by symptoms such as headache, tinnitus, CSF otorrhea, and intracranial venous hypertension. In our study, due to the limitation of sample size, the association between different type of anatomic variations and clinical symptom was not explored. Understanding the nature of variations and the relationship between variations and symptoms is an urgent problem to be solved.

High-resolution black-blood contrast-enhanced MRI may describe the enhancement of the vessel wall and the sac contents (39), and the degree of enhancement is related to the severity of inflammation (40). In this study, a part of anatomic variations and walls of DVSs showed enhancement in T1w black-blood enhanced image. It has already been proved that some indicators of inflammation in serum, such as C reactive protein(CRP), hypersensitive CRP, and erythrocyte sedimentation rate, were raised in patients with IIH (41, 42). Therefore, this finding may be consistent with the hypothesis that inflammation is involved in the pathophysiology of the disease.

In the past, due to the limitations of imaging methods, the understanding of complicated cerebral venous sinus anatomy in patients with IIH was insufficient. This study was, to the best of our knowledge, the first visualization of cerebral venous sinus anatomic variations by using HR-BB contrast-enhanced MRI in patients with IIH. Neuroimaging, such as CTV, MRV, and DSA, has played a significant role in the diagnosis of IIH (43, 44). However, most of these techniques, due to relying on visualization of altered blood flow in the veins, cannot be distinguished from the nature of filing defects (13). For example, AG protruding into the sinus lumen may produce a focal filling defect on MRV that is always confused with intra-sinus BH. HR-BB-MRI is a simple and reliable imaging technology to show the features of lesions, which can be used for reconstruction in axial, sagittal, and coronal positions due to its three-dimensional (3D) acquisition (13, 45). This technique describes the structure of the DVS wall better than other techniques by selectively suppressing signals from the vessel lumen (45). In conclusion, 3D BB contrast-enhanced MRI is a novel MRI sequence for demonstrating and visualizing structures of cerebral dural venous sinus, such as sinus walls, anatomic variabilities, and surrounding tissues in patients with IIH.

There are some limitations in this study. Firstly, due to the rarity of IIH with CVSS, the number of enrolled patients in our study was relatively small. Secondly, although in the real world over men are far less affected by IIH than women, men are half that of females due to the presence of bias in this single-center, continuous study. Thirdly, the imaging time of the current protocol is still long (6–8 min), and further acceleration of data acquisition is highly desirable. Additionally, we did not analyze the relationship between these anatomical variations and the patient's basic information, clinical symptoms and treatment, which will be our focus in the near future. And we also didn't attempt to assess harm-dynamic significance about degree and length of stenosis. It needs further observing in large-scale studies with long-term follow-up to determine if different types of anatomic variation are associated with clinical prognosis. Finally, as a single-center prospective study, we recruited only patients with IIH but not controls. The epidemic data for these anatomic variation of DVSs in enlarged population will be further investigated in future studies.

## Conclusion

HR-BB contrast-enhanced MRI is used to look for any asymmetry of cerebral venous drainage and any abnormal structures impinging on or within DVS in 33 IIH patients with CVSS. Obstruction of CSF outflow in unilateral or bilateral transverse sinus is common in patients with IIH. There are four main types of anatomic variations, including circumscribed stenosis, AG, FS, and BH into DVS. These anatomic variations are one of the main causes of venous reflux disorder by elevating intracranial pressure.

## Data Availability Statement

The original contributions presented in the study are included in the article/supplementary material, further inquiries can be directed to the corresponding author/s.

## Ethics Statement

The study was approved by the Ethics Committee of Beijing Tiantan Hospital. The patients/participants provided their written informed consent to participate in this study.

## Author Contributions

ZZ, JJ, KD, DM, and YW contributed to the conception and design of the study and contributed to the acquisition and analysis of data. YT contributed to drafting the text and preparing the figures. All authors edited and revised the manuscript and approved final submission.

## Funding

This study was supported by grants from the National Natural Science Foundation of China (No. 81825007); Beijing Outstanding Young Scientist Program (No. BJJWZYJH01201910025030); Youth Beijing Scholar Program (No. 010); Beijing Talent Project-Class A: Innovation and Development (No. 2018A12); National Ten-Thousand Talent Plan-Leadership of Scientific and Technological Innovation; National Key R&D Program of China (No. 2017YFC1307900, 2017YFC1307905); Beijing Municipal Administration of Hospitals Incubating Program (No. PX2017009).

## Conflict of Interest

The authors declare that the research was conducted in the absence of any commercial or financial relationships that could be construed as a potential conflict of interest.

## Publisher's Note

All claims expressed in this article are solely those of the authors and do not necessarily represent those of their affiliated organizations, or those of the publisher, the editors and the reviewers. Any product that may be evaluated in this article, or claim that may be made by its manufacturer, is not guaranteed or endorsed by the publisher.
